# Hormonal therapy with megestrol in inoperable hepatocellular carcinoma characterized by variant oestrogen receptors

**DOI:** 10.1054/bjoc.2000.1534

**Published:** 2001-04

**Authors:** E Villa, I Ferretti, A Grottola, P Buttafoco, M Grazia Del Buono, F Giannini, M Manno, H Bertani, A Dugani, F Manenti

**Affiliations:** Department of Internal Medicine, University of Modena, Italy

**Keywords:** variant liver oestrogen receptors transcripts, hepatocellular carcinoma, tamoxifen, megestrol

## Abstract

Variant liver oestrogen receptor transcripts in hepatocellular carcinoma are associated with aggressive clinical course and unresponsiveness to tamoxifen. To evaluate the impact on survival and on tumour growth of megestrol (progestin drug acting at post-receptorial level) we enrolled 45 patients with HCC characterized by variant liver oestrogen receptors in a prospective, randomized study with megestrol vs. placebo. Presence of variant oestrogen receptors was determined by RT/PCR. 24 patients were randomized to no treatment and 21 to therapy with megestrol 160 mg day^−1^. Results were analysed by Kaplan-Meier and Cox methods. Survival of hepatocellular carcinoma characterized by variant oestrogen receptors was extremely poor (median survival 7 months); megestrol significantly improved survival (18 months) (*P* = 0.0090). Tumour growth at one year was significantly slowed down in megestrol-treated patients (*P* = 0.0212). Bilirubin levels, presence of portal thrombosis, HBV aetiology and treatment were identified at univariate analysis as factors significantly associated with survival; at multivariate analysis, only megestrol therapy (*P* = 0.0003), presence of HBV infection (*P* = 0.0009) and presence of portal vein thrombosis (*P* = 0.0051) were factors independently related with survival. (1) Megestrol slows down the aggressive tumour growth of patients with hepatocellular carcinoma characterized by variant estrogen receptors and (2) is also able to favourably influence the course of disease, more than doubling median survival. © 2001 Cancer Research Campaignhttp://www.bjcancer.com


*British Journal of Cancer* (2001) **84**(7), 881–885
© 2001 Cancer Research Campaign
doi: 10.1054/ bjoc.2000.1534, available online at http://www.idealibrary.com on IDEAL®

http://www.bjcancer.com

### Hormonal therapy with megestrol in inoperable hepatocellular carcinoma characterized by variant oestrogen receptors

**E Villa, I Ferretti, A Grottola, P Buttafoco, M Grazia Del Buono, F Giannini, M Manno, H Bertani, A Dugani and F Manenti**

Department of Internal Medicine, University of Modena, Italy

**Summary** Variant liver oestrogen receptor transcripts in hepatocellular carcinoma are associated with aggressive clinical course and unresponsiveness to tamoxifen. To evaluate the impact on survival and on tumour growth of megestrol (progestin drug acting at post-receptorial level) we enrolled 45 patients with HCC characterized by variant liver oestrogen receptors in a prospective, randomized study with megestrol vs. placebo. Presence of variant oestrogen receptors was determined by RT/PCR. 24 patients were randomized to no treatment and 21 to therapy with megestrol 160 mg day$^{-1}$. Results were analysed by Kaplan-Meier and Cox methods. Survival of hepatocellular carcinoma characterized by variant oestrogen receptors was extremely poor (median survival 7 months); megestrol significantly improved survival (18 months) ($P = 0.0090$). Tumour growth at one year was significantly slowed down in megestrol-treated patients ($P = 0.0212$). Bilirubin levels, presence of portal thrombosis, HBV aetiology and treatment were identified at univariate analysis as factors significantly associated with survival; at multivariate analysis, only megestrol therapy ($P = 0.0003$), presence of HBV infection ($P = 0.0009$) and presence of portal vein thrombosis ($P = 0.0051$) were factors independently related with survival. (1) Megestrol slows down the aggressive tumour growth of patients with hepatocellular carcinoma characterized by variant estrogen receptors and (2) is also able to favourably influence the course of disease, more than doubling median survival. © 2001 Cancer Research Campaign http://www.bjcancer.com

**Keywords**: variant liver oestrogen receptors transcripts; hepatocellular carcinoma; tamoxifen; megestrol

Palliative therapy with tamoxifen in inoperable hepatocellular carcinoma has been often used in the past with controversial results (Engstrom et al, 1990; Farinati et al, 1990; Elba et al, 1994; Martin-Cereso et al, 1994): two recent randomized trials of tamoxifen vs. placebo, however, have definitively demonstrated that tamoxifen does not exert any beneficial effect (Castells et al, 1995; Gallo and CLIP study group, 1998).

One possible reason for tamoxifen failure could reside in the presence, in a substantial percentage of patients with hepatocellular carcinoma (HCC), of variant liver oestrogen receptors (Villa et al, 1998): these receptors derive from an exon 5-deleted oestrogen receptor transcript which gives rise to an oestrogen receptor altered in the hormone-binding domain (*v*ER), capable, however, of maintaining a constitutive transcriptional activity (Fuqua et al, 1991). Tumours characterized by this receptor have significantly shorter doubling time in comparison with tumours characterized by wild-type ER (*wt*ER) transcripts, a higher clinical aggressiveness and are unresponsive to tamoxifen, as this drug acts blocking the receptor protein by competing for the hormone binding site and requires an intact receptor to exert its action (Villa et al, 1995, 1996a, 1996b). A small open trial in 8 patients suggested that in these patients, a progestin drug like megestrol (which exerts its action at post-receptorial level) might represent a better therapeutic option than tamoxifen (Villa et al, 1996a, 1996b).

*Received 10 December 1999*
*Revised 1 August 2000*
*Accepted 13 September 2000*

*Correspondence to:* E Villa

We have therefore planned a prospective, randomized, placebo-controlled study in the attempt to define whether antihormonal therapy with megestrol may slow down tumour growth and improve survival in HCC patients with variant ERs. As antihormonal therapy cannot be considered a radical therapeutical measure, only patients with inoperable HCC were included in the study.

### MATERIALS AND METHODS

### Selection of patients and treatment protocol

This prospective, randomized, controlled study was carried out at the University of Modena between September 1995 and September 1998; enrolment of patients ended in July 1997. Patients were eligible for the study if they had inoperable HCC, i.e. if clinical conditions, size (solitary tumour larger than 5 cm) or number (3 or more nodules) of the neoplastic lesions precluded surgery (Castells et al, 1993), liver transplant (Mazzaferro et al, 1996) or percutaneous ethanol injection (Vilana et al, 1992). 9 patients, when first seen, had solitary lesions smaller than 5 centimetres: however, extremely rapid increase of the size of the lesion (8 patients) and refusal of any type of therapy ($n = 1$) precluded any attempt of radical treatment and patients could therefore be enrolled in this study. Additional criteria for entry was a performance score of at least 70 on the Karnofsky scale. Demonstrable extrahepatic metastases by total body CT or bone scan and previous or simultaneous secondary malignant disease excluded patients from the protocol. The study was approved by the Institutional Ethical Committee of the University of Modena; patients gave informed written consent before enrolment in the study.

**Table 1** Comparison of the clinical and demographic features at enrolment in the 45 patients studied, divided according to treatment

| | Whole group (%) | Controls (24) (%) | Megestrol (21) (%) | Level of significance (*P*) |
|---|---|---|---|---|
| Age (mean ± SD)[a] | 62 ± 11 | 60 ± 11 | 63 ± 8 | 0.724 |
| Males/Females | 36/9 | 22/2 | 14/7 | 0.061 |
| Aetiology | | | | |
| HBsAg (%) | 29 (64.4) | 9 (37.5) | 6 (28.5) | |
| AntiHCV (%) | 15 (33.3) | 15 (62.5) | 14 (66.6) | 0.487 |
| Alcohol (%) | 1 (2.2) | – | 1 (4.7) | |
| Child-Pugh | | | | |
| A | 21 (46.6) | 10 (41.6) | 11 (52.3) | |
| B | 13 (28.8) | 7 (29.1) | 6 (28.5) | 0.689 |
| C | 10 (24.4) | 7 (29.1) | 4 (19.0) | |
| Basal tumour volume ($cm^3$)[a] | 132 ± 251 | 158 ± 293 | 104 ± 203 | 0.334 |
| Histology | | | | |
| Well differentiated | 23 (51.1) | 14 | 9 | |
| Moderately differentiated | 11 (24.4) | 5 | 6 | 0.604 |
| Poorly differentiated | 6 (13.3) | 2 | 4 | |
| Unknown | 5 (11.1) | 3 | 2 | |
| Portal vein thrombosis | 5 (11.1) | 4 | 1 | 0.352 |
| Tumour stage | | | | |
| Single nodule <5 cm | 9 (20.0) | 6 | 3 | |
| Single nodule >5 cm | 15 (33.3) | 7 | 8 | 0.632 |
| Multiple nodules or diffuse | 21 (46.7) | 11 | 10 | |
| Albumin ($g\ l^{-1}$)[a] | 3.2 ± 0.5 | 3.1 ± 0.4 | 3.3 ± 0.4 | 0.098 |
| Alkaline phosphatase ($IU\ l^{-1}$)[a] | 365 ± 144 | 334 ± 212 | 332 ± 144 | 0.657 |
| Bilirubin ($mg\ dl^{-1}$)[a] | 1.6 ± 0.9 | 1.7 ± 1.4 | 1.6 ± 1.9 | 0.368 |
| GGT ($IU\ l^{-1}$)[a] | 137 ± 130 | 163 ± 175 | 157 ± 99 | 0.045 |
| Prothrombin time (% activity)[a] | 76 ± 18 | 77 ± 22 | 74 ± 15 | 0.973 |
| AFP (<100/100–500/>500) | 31/3/6 | 15/1/4 | 16/2/2 | 0.738 |

[a] Mann-Whitney test.

Patients underwent ultrasound-assisted liver biopsy as a part of routine histopathological diagnosis and for evaluation of type of ER transcript. Written informed consent was obtained from each patient for the use of part of the biopsy for purpose other than diagnostic histopathological investigation. ER transcripts were determined by RT/PCR as already described (Villa et al, 1995).

Demographic characteristics, aetiology and severity of cirrhosis (evaluated by Child-Pugh score) were assessed in each patient (Table 1).

The inclusion criteria were met by 45 patients, who were randomly assigned, by sealed envelope method, either to the control group or to hormonal treatment with megestrol 160 mg $day^{-1}$.

### Patients follow up

In view of the advanced stage of disease in these patients, primary goal of the study was stabilization of disease and improvement of survival; treatment was stopped only when relevant side-effects occurred. All adverse effects were recorded, whether or not they were considered as drug-related. Patients, both treated and controls, were seen every 3 months, unless relevant side effects or complications occurred, in which case they were admitted to hospital. At each visit, medical history was taken and physical examination was performed. Laboratory tests (blood count, alfaetoprotein (AFP), bilirubin, prothrombin time, alkaline phosphatase, gammaglutam transpeptidase (GGT), serum albumin) were carried out every 3 months. Magnetic resonance was performed at month 3, 12 and 24, as already described (Kaplan and Meier, 1958).

### Virological tests

HBsAg was determined by Ausria II (Abbott Laboratories, Chicago, IL). AntiHCV was determined by third generation ELISA (Ortho Diagnostic Systems, Raritan, NJ).

### Statistical analysis

Baseline data of patients are reported as mean ± standard deviation (SD) or median and ranges. Qualitative variables were compared with chi square test or with Fischer exact test when appropriate while quantitative variables were compared with Mann-Whitney U test. All *P* values were two-tailed and level of significance was set to 0.05.

The univariate analysis to identify predictors of survival was performed by the Kaplan-Meier method (1958) and compared by the Mantel log-rank test. Data were censored at the time of last visit. 16 variables were assessed: age, AFP, alkaline phosphatase, ascites, bilirubin, Child-Pugh score, encephalopathy, aetiology, gamma-glutamyltranspeptidase (GGT), histology of tumour, increment of tumour volume at month 3, megestrol treatment, platelets count, presence of portal vein thrombosis, previous variceal haemorrhage and sex. For continous variables the cut-off was set at the median value. All variables were subsequently analysed by Cox's regression method (Cox, 1972).

Time to tumour progression was analysed, according to WHO criteria, using the method of Kaplan and Meier (1958). Progression-free patients were censored at death. User-defined missing values were treated as missing.

All calculations were performed with the SPSS/PC plus 2 statistical package (SPSS, Chicago, IL).

 *© 2001 Cancer Research Campaign*

### RESULTS

### Clinical and laboratory data

135 patients were diagnosed in our unit in the period 1995–1997 as having HCC; of them, in 14 patients (33.3%) (36 males, 9 females) had variant ERs transcripts demonstrated in the tumour and were therefore enrolled in the study. All patients had cirrhosis of the liver. Clinical and demographic data of the patients at enrolment are listed in Table 1. Patients in the treated and untreated group were similar in regard to age, Child-Pugh score, volume of tumour mass at baseline, histology, presence of portal vein thrombosis, stage of the tumour and biochemical data; despite higher number of females in treated group, this difference did not reach significance (*P* = 0.061. Fischer's exact test).

Both treated patients and controls were regularly seen in the outpatient clinic every 3 months; follow-up schedule was exactly the same and no dropouts occurred.

24 patients were randomized to no treatment while 21 were received megestrol 160 mg day$^{-1}$. No violation of randomization occurred.

### Survival analysis

Death occurred in 34 out of 45 patients (75.5%). At the end of follow-up, the overall median survival was 14 months (95% CI 9.28–18.72 months). The overall actuarial probability of survival at year 1, 2, 3 and 4 was 52, 19, 15 and 9%, respectively.

When the patients were divided by treatment, the median survival in untreated patients was 7 months (95% CI 3.01–10.99 months) versus 18 months (95% CI 13.47–22.53 months) in megestrol-treated patients (*P* = 0.0090) (Figure 1). The overall actuarial probability of survival at year 1, 2, 3 and 4 was 29, 15, 15 and 4% in untreated patients and 83, 25, 15 and 15% in megestrol-treated patients.

Of the 16 variables assessed at univariate analysis (Table 2), 4 were found to be significantly related with survival: megestrol treatment (*P* = 0.0090); bilirubin levels (*P* = 0.0451); HBV-related aetiology (*P* = 0.0453); presence of portal vein thrombosis (*P* = 0.0246). Evaluation of treatment effect after adjustment for patients' sex evidenced a significant difference in favour of female patients (males: treated vs. controls: median survival 18 vs. 7 months; 95% CI: 6.36–29.64 and 3.19–10.81 respectively; females: treated vs. controls median survival 17 vs. 3 months; 95% CI 15.40–18.60 and 0 respectively) (*P* = 0.0078).

All variables from all 45 patients were included in multivariate analysis. Megestrol treatment (hazard ratio: 0.2098; 95% CI: 0.0894–0.4923; *P* = 0.0003), HBV-related aetiology (hazard ratio: 4.3221; 95% CI: 1.9255–9.7015; *P* = 0.0009) and presence of portal vein thrombosis (hazard ratio: 5.7395; 95% CI: 1.6850–19.5504; *P* = 0.0051) were independent predictive factors of survival.

### Tumour growth

Mean tumour mass at baseline was not significantly different between treated and untreated patients (untreated: 158 ± 293 cm$^3$; treated 140 ± 203 cm$^3$) (*P* = 0.334). Analysis to time of first tumour growth by Kaplan-Meier showed time to progression to be significantly increased in the treated group (median time 22 months for megestrol-treated patients vs. 9 months for controls, log rank 5,33, *P* = 0.0212) (Figure 2).

### Side effects

Treatment was very well tolerated by the majority of the patients. Increase in appetite and in weight occurred in 15 (71.4%) and 13 (61.9%) out of 21 treated patients respectively in comparison with none of the untreated patients.

One patient (4.1%) in the treated group and one (4.7%) in the control group developed deep vein thrombosis. One of the female patients, aged 65, treated with megestrol, experienced moderate vaginal spotting, which resolved spontaneously, without the need to stop therapy.

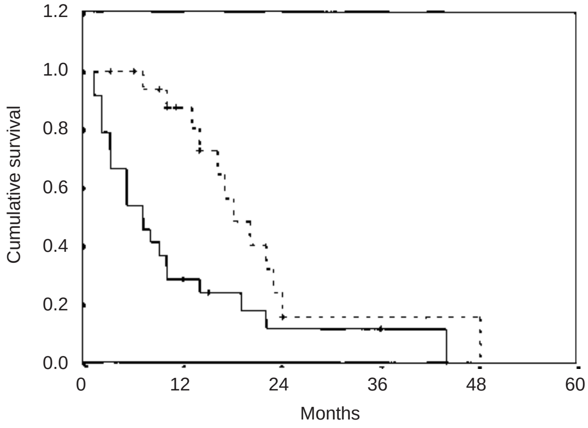

| Number at risk | | | | | |
|---|---|---|---|---|---|
| Megestrol | 21 | 12 | 3 | 2 | 2 |
| Control | 24 | 7 | 2 | 1 | 0 |

**Figure 1** Kaplan-Meier estimated probability of survival in treated patients and controls (*P* = 0.0090 by Log rank test).

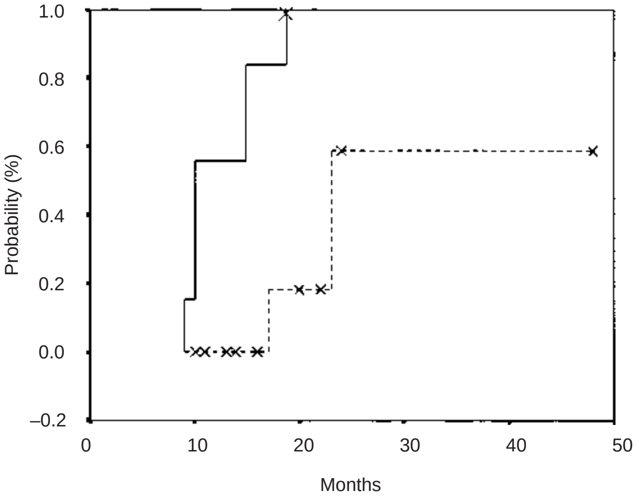

**Figure 2** Actuarial probability of growth of tumour mass. Increase of tumour volume was significantly different between control and patients treated with megestrol (*P* = 0.0212 by Log rank test).

© 2001 Cancer Research Campaign

**Table 2** Evaluation of the prognostic value of variable by Kaplan-Meier analysis (comparison by Log rank test)

| Variable | | Number of patients | Number of deaths | Median survival (mo) | 95% Confidence Interval | Significance (*P*) |
|---|---|---|---|---|---|---|
| Variables with prognostic significance | | | | | | |
| Megestrol treatment | | | | | | |
| | Yes | 21 | 13 | 18 | 13.47–22.53 | 0.0090 |
| | No | 24 | 21 | 7 | 3.01–10.99 | |
| Bilirubin | ≤ 1.0 mg% | 35 | 25 | 14 | 3.69–24.31 | 0.0451 |
| | > 1.0 mg% | 10 | 9 | 5 | 0–15.85 | |
| Aetiology | HBV | 16 | 14 | 7 | 1.14–12.86 | 0.0453 |
| | HCV | 29 | 20 | 17 | 12.08–21.92 | |
| Portal vein thrombosis | | | | | | |
| No | | 40 | 30 | 14 | 6.71–21.29 | 0.0246 |
| Yes | | 5 | 4 | 2 | 0.93–3.07 | |
| Variables without prognostic significance | | | | | | |
| Age | ≤ 64 years | 22 | 19 | 13 | 0.27–25.73 | 0.9732 |
| | > 64 years | 23 | 15 | 14 | 9.42–18.58 | |
| AFP | ≤ 21 ng ml$^{-1}$ | 20 | 11 | 19 | 9.49–28.51 | 0.3406 |
| | > 21 ng ml$^{-1}$ | 20 | 18 | 13 | 6.13–19.87 | |
| Alk. Phosph. | ≤ 280 IU l$^{-1}$ | 14 | 8 | 16 | 6.28–25.72 | 0.1813 |
| | > 280 IU l$^{-1}$ | 31 | 26 | 10 | 2.85–17.15 | |
| Ascites | Absent | 32 | 24 | 13 | 6.6–21.46 | 0.7968 |
| | Moderate | 9 | 6 | 14 | 2.68–25.32 | |
| | Severe | 4 | 4 | 7 | 0.14–13.86 | |
| Child-Pugh | A | 22 | 17 | 10 | 3.48–16.52 | |
| | B | 13 | 9 | 14 | 7.0–20.91 | 0.8848 |
| | C | 10 | 8 | 10 | 0.70–19.30 | |
| Encephalopathy | | | | | | |
| | Absent | 39 | 29 | 13 | 8.70–17.30 | 0.4143 |
| | Moderate | 6 | 5 | 19 | 0–42.52 | |
| GGT | < 86 IU l$^{-1}$ | 21 | 16 | 14 | 4.08–23.92 | 0.5236 |
| | > 86 IU l$^{-1}$ | 24 | 18 | 13 | 4.28–21.72 | |
| Histology | | | | | | |
| Well differentiated | | 23 | 19 | 13 | 8.60–17.40 | |
| Moderately different. | | 11 | 6 | 24 | 6.15–41.95 | 0.4023 |
| Poorly differentiated | | 6 | 5 | 7 | 0.00–25.00 | |
| Unknown | | 5 | 5 | 24 | 4.92–43.08 | |
| % Increment of tumour mass at month 3 | | | | | | |
| Less than 55% | | 18 | 14 | 14 | 3.40–24.60 | 0.6198 |
| More than 55% | | 20 | 15 | 10 | 0.97–19.03 | |
| Platelet count | | | | | | |
| ≤ 100.000 mmc | | 21 | 15 | 17 | 0.48–33.52 | 0.8231 |
| > 100.000 mmc | | 24 | 20 | 14 | 12.17–15.83 | |
| Previous variceal haemorrhage | | 40 | 30 | 13 | 6.98–19.02 | 0.2292 |
| No | | 5 | 4 | 14 | 0.00–34.28 | |
| Yes | | | | | | |
| Sex | Males | 36 | 29 | 10 | 4.61–15.39 | 0.8505 |
| | Females | 9 | 5 | 16 | 8.75–23.25 | |

### DISCUSSION

HCC patients whose tumours are characterized by variant ERs have a definitively more aggressive disease than patients with wild-type ER: tumours grow very rapidly and survival is significantly shorter than in patients in whom liver wild-type receptors are present (Villa et al, 1997). Indeed, median survival of untreated patients in the present series was 7 months, despite the fact that their cirrhotic condition at enrolment was not very advanced, as indicated by the preserved liver function and by the lack of correlation between survival and signs of severity of disease like Child-Pugh score ($P$ = 8848), presence of ascites ($P$ = 0.7968) or of encephalopathy ($P$ = 0.4143); paradoxically, subjects with moderate encephalopathy or moderate ascites had better survival, perhaps as they had slightly slower course of disease. Megestrol was able to favourably influence such severe course, significantly improving survival, which increased from 7 to 18 months, and slowing down tumour growth.

Our results are in contrast with those by Chao et al (1991) who did not find any favourable effect in 32 patients with inoperable HCC treated with the same dosage of megestrol used in our study. The authors, however, defined as goal of the study the disappearance or at least the reduction of more than 50% of the tumour mass, which were not achieved. As the study was not controlled, the authors did not have the chance to evidence a possible improvement in survival between treated and control group, which was instead the most notable result in our series. Indeed, also in none of our patients was there regression of tumour mass but, in treated patients tumour progression was significantly slower and survival longer than in untreated patients. On the other hand, one should recognize the fact that in both studies, the patients under investigation were characterized by very advanced disease and in

*© 2001 Cancer Research Campaign*

the study by Chao et al also by previous other therapies in more than 60% of patients, therefore with the worst characteristics in terms of possible response to therapy.

Our results suggest also that the sensitivity of HCC patients to megestrol is probably short-lived: the comparison of the cumulative proportions of survival in treated and untreated patients shows that there is a net improvement in the first year (83% treated vs. 29% untreated) but afterwards the difference is no longer so striking and the survival curves of treated and untreated patients converge. The effect of megestrol is likely not powerful enough to stop tumour progression but it is able to counteract tumour growth for a period sufficient to improve significantly the course of the disease. On the other hand, no other therapeutic regimens, evaluated in a controlled fashion, have so far obtained definite good results, especially concerning survival, in this type of patients (Simonetti et al, 1997).

Both univariate and multivariate analysis indicated, apart from treatment, HBV-related aetiology as one of the factors negatively associated with survival. The severe prognosis of patients with HBV-related disease in comparison with the HCV-positive ones has been already recognized (Zaman et al, 1985; Villa et al, 1988). It is possible that in the present series this finding may be due to the preselection of patients with HCC characterized by variant ER receptors transcripts: these subjects are in fact significantly more often HBsAg-positive than HCV-positive (Villa et al, 1998). On the other hand, the capability of HBV to integrate into host genome might facilitate the occurrence of alternative splicing events of ER gene with onset of variant ERs.

We have not observed a difference in overall survival between males and females; however hormonal manipulation seemed to exert a better effect in females than in males despite the fact that these tumours were all characterized by receptors, like variant ERs, less sensitive to hormonal manipulation than classical wild-type ERs (Fuqua, et al, 1991; Villa et al, 1996a).

In conclusion, our results show that megestrol in patients with HCC characterized by variant ERs, i.e. in those patients with rapidly progressive disease, is able to remarkably increase survival. The effect is not sufficient to cure the disease but the increased survival together with the very good tolerance of the drug, the improvement of feeling of well being and the lack of serious side effects make a trial with this drug more than warranted. Future efforts should be addressed to identify more powerful drugs which could interfere more efficiently with the aggressive course of disease in HCC patients with variant ERs.

### ACKNOWLEDGEMENTS

This work was supported by grants ‘60%’ of the University of Modena, ‘40%’ of the Ministry of University and of Scientific and Technological Research (MURST), Progetti di ricerca con ricaduta assistenziale Azienda Policlinico di Modena and Associazione Italiana per la Ricerca sul Cancro (AIRC).

### REFERENCES

Castells A, Bruix J, Bru C, Fuster J, Vilana R, Navasa M, Ayuso C, Boix L, Visa J and Rodes J (1993). Treatment of small hepatocellular carcinoma in cirrhotic patients: a cohort study comparing surgical resection and percutaneous ethanol injection. *Hepatology* **18**: 1121–1126

Castells A, Bruix J, Bru C, Ayuso C, Roca M, Boix L, Vilana R and Rodes J (1995) Treatment of hepatocellular carcinoma with tamoxifen: a double-blind trial in 120 patients. *Gastroenterology* **109**: 917–922

Chao Y, Chan W-K, Wang S-S, Lai KH, Chi CW, Lin CY, Chan A, Whang Peng-J, Lui WY and Lee SD (1991). Phase II study of megestrol acetate in the treatment of hepatocellular carcinoma. *J Hepatol Gastroenterol* **12**: 277–281

Cox DR (1972) Regression models and life tables (with discussion). *J R Stat Soc B* **34**: 187–220

Elba S, Giannuzzi V, Misciagna G and Manghisi OG (1994) Randomized controlled trial of tamoxifen versus placebo in inoperable hepatocellular carcinoma. *It J Gastroenterol* **26**: 66–68

Engstrom PF, Levin B, Moertel CG and Schutt A (1990) A Phase II trial in hepatocellular carcinoma. *Cancer* **65**: 2641–2643

Farinati F, Salvagnini M, De Maria N, Fornasiero A, Chiaramonte M, Rossaro L and Naccarato R (1990) Unresectable hepatocellular carcinoma. A prospective controlled trial with tamoxifen. *J Hepatol* **11**: 297–301

Fuqua SAW, Fitzgerald SD, Chamness GC, Tandon AK, McDonnell DP, Nawaz Z, O’Malley BW and McGuire WL (1991) Variant human breast tumor estrogen receptor with constitutive transcriptional activity. *Cancer Res* **51**: 105–109

Gallo C and CLIP study group (1998). Tamoxifen in the treatment of hepatocellular carcinoma: a randomised controlled trial. *Lancet* **352**: 17–20

Kaplan EL and Meier P (1958) Non-parametric estimation for incomplete observation. *Am Stat Assoc* **53**: 457–481

Martinez-Cerezo FJ, Tomas A, Donoso L, Enriquez-J, Guarner C, Balanzo J, Martinez-Nogueras A and Vilardell F (1994) Controlled trial of tamoxifen in patients with advanced hepatocellular carcinoma. *J Hepat* **20**: 702–706

Mazzaferro V, Regalia E, Doci R, Andreola S; Pulvirenti A, Bozzetti F, Montalto F, Ammatuna M, Morabito A and Gennari L (1996) Liver transplantation for the treatment of small hepatocellular carcinomas in patients with cirrhosis. *N Engl J Med* **334**: 693–699

Simonetti RG, Liberati A, Angiolini C and Pagliaro L (1997). Treatment of hepatocellular carcinoma: a systematic review of randomized controlled trials. *Ann Oncol* **8**: 117–136

Vilana R, Bruix J, Bru C, Ayuso C, Sole M and Rodes J (1992) Tumor size determines the efficacy of percutaneous ethanol injection for treatment of small hepatocellular carcinoma. *Hepatology* **16**: 353–357

Villa E, Baldini, G, Pasquinelli C, Melegari M, Cariani E, Di-Chirico G and Manenti F (1988). Risk factors for hepatocellular carcinoma in Italy: HBV virus, alcohol, male sex. *Cancer* **62**: 611–615

Villa E, Camellini L, Dugani A, Zucchi F, Grottola A, Merighi A, Buttafoco P, Losi L and Manenti F (1995) Variant estrogen receptor messenger RNA species detected in human primary hepatocellular carcinoma. *Cancer Res* **55**: 498–500

Villa E, Camellini L, Dugani A, Buttafoco P, Grottola A and Manenti F (1996a) Variant liver estrogen receptors and response to tamoxifen. *Gastroenterology (Letter)* **111**: 271–272

Villa E, Dugani A, Fantoni E, Camellini L, Buttafoco P, Grottola A, Pompei G, De-Santis M, Ferrari A and Manenti F (1996b). Type of estrogen receptor determines response to antiestrogen therapy in hepatocellular carcinoma. *Cancer Res* **56**: 3883–3885

Villa E, Fantoni E, Moles A, Camellini L, Buttafoco P, Grottola A, Ferretti I, Scarcelli A and Manenti F (1997). Survival of patients with hepatocellular carcinoma is determined by type of liver estrogen receptors. *Hepatology* **26**: 247A

Villa E, Dugani A, Moles A, Camellini L, Grottola A, Buttafoco P, Merighi A, Ferretti I, Esposito P, Miglioli L, Bagni A, Troisi R, De-Hemptinne B, Praet M, Callea F and Manenti F (1998). Variant liver estrogen receptor transcripts already occur at an early stage of chronic liver disease. *Hepatology* **27**: 983–988

Zaman SN, Melia WM, Johnson RD, Portmann BC, Johnson PJ and Williams R (1985) Risk factors in development of hepatocellular carcinoma in cirrhosis: prospective study of 613 patients. *Lancet* **1**: 1357–1360

© 2001 Cancer Research Campaign 
